# Correction: Utilizing the bioorthogonal base-pairing system of l-DNA to design ideal DNA nanocarriers for enhanced delivery of nucleic acid cargos

**DOI:** 10.1039/c5sc90009c

**Published:** 2015-01-23

**Authors:** Kyoung-Ran Kim, Taemin Lee, Byeong-Su Kim, Dae-Ro Ahn

**Affiliations:** a Center for Theragnosis , Biomedical Research Institute , Korea Institute of Science and Technology , Hwarangno 14-gil 5, Seongbuk-gu , Seoul 136-791 , Republic of Korea . Email: drahn@kist.re.kr ; Fax: +82 2 958 5909 ; Tel: +82 2 958 6645; b KIST campus , University of Science and Technology (UST-KIST) , Hwarangno 14-gil 5, Seongbuk-gu , Seoul 136-791 , Republic of Korea; c Interdisciplinary School of Green Energy , Ulsan National Institute of Science and Engineering (UNIST) , Ulsan 689-798 , Republic of Korea; d Department of Chemistry , College of Science , Yonsei University , Republic of Korea

## Abstract

Correction for ‘Utilizing the bioorthogonal base-pairing system of l-DNA to design ideal DNA nanocarriers for enhanced delivery of nucleic acid cargos’ by Kyoung-Ran Kim *et al.*, *Chem. Sci.*, 2014, **5**, 1533–1537.



## 

In the paper, the figure legend to Fig. 1d written as “(d) CD spectra of d-Td (blue) and l-Td (red)” should be corrected as follows: “(d) CD spectra of d-Td (red) and l-Td (blue)”.

The Royal Society of Chemistry apologises for these errors and any consequent inconvenience to authors and readers.

